# Regular or Irregular Breakfast Skipping Suppresses the Vascular Endothelial Function of the Brachial Artery

**DOI:** 10.3390/nu17203244

**Published:** 2025-10-15

**Authors:** Hideaki Kashima, Yui Morinaka, Kano Endo, Mizuki Sugimoto, Naho Nagao, Ryota Mabuchi, Masako Yamaoka Endo, Naomi Kashima, Yasuhiko Kitadai, Akira Miura, Yoshiyuki Fukuba

**Affiliations:** 1 Department of Exercise Science and Physiology, School of Health Sciences, Prefectural University of Hiroshima, Hiroshima 734-8558, Japan; 2 Faculty of Bioresource Science, Prefectural University of Hiroshima, Shobara 727-0023, Japan; 3 Faculty of Health Sciences, Hiroshima Shudo University, Hiroshima 731-3195, Japan; 4 Faculty of Health and Sports Sciences, Hiroshima International University, Hiroshima 739-2695, Japan

**Keywords:** breakfast skipping, endothelial function, free fatty acid, insulin, postprandial hyperglycemia

## Abstract

Background: Habitual breakfast skipping is associated with an increased risk of cardiovascular and cardiometabolic diseases. However, the effects of skipping breakfast regularly versus irregularly on vascular endothelial function (VEF), a key marker of cardiovascular health, remain unclear. This study aimed to investigate the effects of eight-Day regular or irregular breakfast skipping on brachial artery VEF in healthy habitual breakfast eaters using a three-condition, randomized controlled crossover trial. Methods: Ten young healthy adults (seven females, three males) completed three randomized nine-Day trials: (1) Eat (three meals per day), (2) Skip (breakfast skipped on days 1–8, consumed on Day 9), and (3) Eat/Skip (alternating breakfast consumption and skipping). Flow-mediated dilation (FMD) of the right brachial artery was assessed at 7:45–55 am on days 1, 2, 5, and 9, expressed as the percentage change in the brachial artery diameter normalized to the shear rate area under the curve (Δ%FMD/SR_AUC_). Blood samples were collected before and 30 min after breakfast or lunch for glucose, insulin, free fatty acids, and triglyceride analyses. Insulin resistance was estimated using the homeostasis model assessment of insulin resistance calculated from fasting glucose and fasting insulin values. Objective measurements of sleep, physical activity, and continuous glucose monitoring were obtained. Results: On Day 9, the Skip and Eat/Skip trials had significantly lower %FMD/SR_AUC_ and significantly higher levels of fasting plasma insulin than the Eat trial. Exploratory analyses within the Skip and Eat/Skip trials suggested a weak negative association between changes in %FMD/SR_AUC_ and fasting blood glucose and insulin from day 1 to day 9. Conclusions: These findings suggest that both regular and irregular breakfast skipping may impair early morning VEF, possibly through alterations in glucose metabolism, whereas regular breakfast consumption may help preserve VEF and support cardiovascular health. Clinical Trial Registry: Clinical Trial Registry: University Hospital Medical Information Network (UMIN000053117, registered 20 December 2023).

## 1. Introduction

Generally, endothelial dysfunction initiates atherosclerosis, a major contributor to cardiovascular disease [1]. Endothelial nitric oxide helps maintain vascular homeostasis through vasodilatory and antiatherosclerotic actions [2]. Therefore, preserving vascular endothelial function (VEF) is critical for preventing cardiovascular disease. Flow-mediated dilatation (FMD) of the brachial artery is a widely accepted, noninvasive index of VEF [3,4], given that impaired FMD is associated with increased cardiovascular morbidity and mortality [5,6].

According to several epidemiological studies, habitual breakfast skipping is an independent risk factor for cardiovascular disease [7,8,9,10,11]. An observational study also reported that individuals who habitually skip breakfast exhibit greater arterial stiffness than those who regularly eat breakfast, even after adjusting for potential confounders and cardiovascular risk factors [12]. Despite this well-documented association, the underlying physiological mechanisms, particularly those related to vascular function, remain unclear. Given that VEF is an early marker of cardiovascular dysfunction, elucidating how breakfast habits influence VEF is crucial for both public health and clinical practice.

In healthy adults, skipping breakfast causes a higher glycemic response to lunch than eating breakfast [13,14,15]. Postprandial hyperglycemia acutely suppresses VEF through oxidative stress [16,17,18,19]. One of the mechanisms underlying postprandial hyperglycemia after breakfast skipping may be the sustained increase in plasma free fatty acids (FFAs) by lunch [13,14]. Specifically, elevated FFAs inhibit insulin-dependent glucose uptake in skeletal muscle and impair insulin-dependent nitric oxide production in the endothelium [20,21]. Recently, we demonstrated that one-time breakfast skipping significantly suppressed postlunch VEF in healthy adults and that the degree of impairment negatively correlated with postprandial glucose levels [22]. However, this finding is based primarily on single-day interventions. Thus, the cumulative vascular impact of repeated breakfast skipping remains unclear.

Currently, the metabolic consequences of skipping breakfast for several days in a controlled meal situation are insufficiently studied. In the study of Ogata et al. [23], 24 h interstitial glucose levels elevated after 6 days of breakfast skipping when the participants remained in a metabolic chamber [23]. Thus, given that postprandial hyperglycemia may impair VEF (16–18), repeated breakfast skipping might lead to VEF decline.

Interestingly, Ogata et al. [23] also observed no difference in postlunch glucose response on Day 2 after breakfast skipping, suggesting a potential “metabolic adaptation.” However, their breakfast-skipping trial included a higher energy intake at lunch [23], complicating the interpretation of whether this adaptation was physiological or nutritional. Furthermore, their study lacked detailed blood analyses (e.g., insulin and FFAs); thus, mechanistic links to postprandial glycemic control could not be elucidated.

Critically, no previous study has directly compared the vascular effects of regular (daily) and irregular (alternate-day) breakfast skipping, despite the fact that real-world eating patterns often vary. Therefore, this study aimed to investigate the effects of these two breakfast-skipping patterns for 8 days on VEF in the brachial artery, glucose, insulin, and lipid profiles in healthy young adults under controlled dietary trials.

## 2. Materials and Methods

### 2.1. Participants

Eleven individuals voluntarily participated from Prefectural University of Hiroshima via campus-wide emails and posters between January and May 2024, but one was eventually excluded because of insulin resistance (homeostasis model assessment-insulin resistance, HOMA-IR > 2.5), which was calculated as follows: fasting plasma insulin (µU/mL) × fasting blood glucose (mg/dL)/405 [24]. Ultimately, 10 healthy young adults (7 females and 3 males; age: 20 ± 1 years; height: 160 ± 4 cm; body weight: 54 ± 5 kg; body mass index [BMI]: 21 ± 2 kg/m^2^) were included for the analysis. All participants reportedly ate breakfast regularly (≥5 days/week), were nonsmokers, normotensive, free of gastrointestinal symptoms, and not taking any medications, and had no history of cardiovascular or metabolic disease. None of them had food allergies, and all met the insulin sensitivity inclusion criterion (HOMA-IR ≤ 2.5). The required sample size was determined using G*Power software (version 3.1.9.2) according to prior data from Ogata et al. (23), who assessed the average 24 h glucose levels in healthy young men under breakfast skipping versus breakfast consumption trials over 6 consecutive days. On the basis of an effect size of 1.444 (breakfast consumption: 90.5 ± 6.5 mg/dL; breakfast skipping: 94.0 ± 7.5 mg/dL; r = 0.95), with α = 0.05 and power = 0.80, the estimated minimum sample size was eight. Considering potential dropouts, we set the target sample size at 10. All procedures conformed to the Declaration of Helsinki, with approval from the Ethics Committee of the Prefectural University of Hiroshima (approval number: 23HH0011). All participants provided written informed consent. The trial was registered at the University Hospital Medical Information Network (UMIN000053117) on 15 December 2023.

### 2.2. Main Trial Sessions

**Figure 1:**
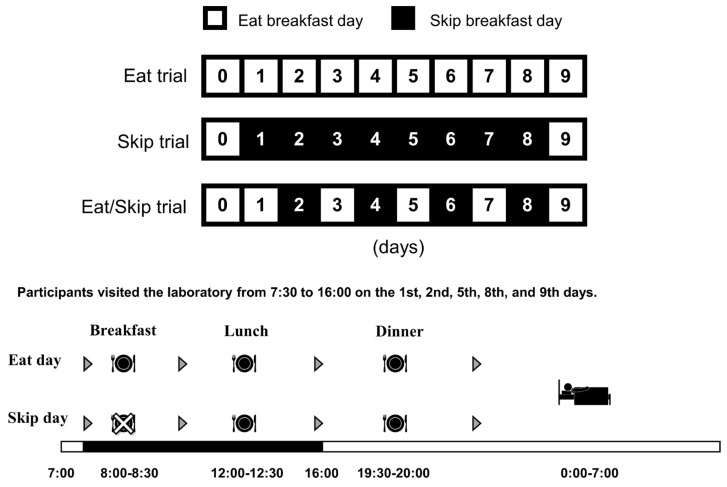
Overview of the experimental design and meal schedule across the three trials (Eat, Skip, and Eat/Skip). Each intervention lasted 9 days. In the Eat trial, participants consumed breakfast, lunch, and dinner at fixed times (8:00–8:30, 12:00–12:30, and 19:30–20:00, respectively) throughout the intervention. In the Skip trial, participants skipped breakfast from days 1 to 8, consuming only lunch and dinner, but breakfast was reintroduced on day 9. In the Eat/Skip trial, participants alternately consumed and skipped breakfast from days 1 to 9. Laboratory measurements were conducted before and after breakfast and lunch on days 1, 2, 5, 8, and 9 in all trials.

This crossover controlled study comprised three laboratory trials. In the first trial, we randomly assigned the participants to one of the three groups by using computer-generated random numbers: Eat (*n* = 4), Skip (*n* = 3), and Eat/Skip (*n* = 3). A washout period of at least one month was maintained between trials. In each experimental protocol, which lasted 9 days, the participants underwent laboratory measurements before and after breakfast and lunch on days 1, 2, 5, 8, and 9. In the breakfast consumption trial (Eat), they consumed three meals per day at consistent times for 9 days (Figure 1). In the breakfast-skipping trial (Skip), they ate only lunch and dinner from days 1 to 8 but had breakfast on Day 9. In the irregular trial (Eat/Skip), they ate and skipped breakfast alternately from days 1 to 9. Breakfast on days when meals were skipped in the Skip and Eat/Skip trials was split evenly between lunch and dinner. From the day before the experiment until Day 9, we scheduled breakfast (or its skipping) between 8:00 and 8:30, lunch between 12:00 and 12:30, and dinner between 19:30 and 20:00, and all meals were standardized. Table 1 presents the nutritional composition of the five meal types (A–E). Male participants received an additional 200 kcal liquid containing macronutrients (Calorie Mate Jelly [energy: 200 kcal; protein: 8.2 g; fat: 4.4 g; carbohydrates: 31.2 g; salt: 0.17 g]; Otsuka Pharmaceutical Co., Ltd., Saga, Japan) per meal. On the breakfast-skipped days in both the Skip and Eat/Skip trials, the granola that was originally designated for breakfast was reallocated to dinner, and the remaining meals of the breakfast meal were incorporated into lunch. We served meal A on the day before the experiment and on Day 5, meal B on days 2 and 9, and meal D on days 3 and 8. On the laboratory measurement days (days 1, 2, 5, 8, and 9), the participants ate their breakfast and lunch in the laboratory and their dinner and other meals at their homes. We instructed them to refrain from performing strenuous exercises, as well as consuming alcohol and caffeine, throughout the experimental period; they were restricted to water or barley tea. Additionally, bedtime was regulated between 23:00 and 24:00.

Upon arriving at the laboratory at 7:30 for measurement, the participants rested in a supine position in a quiet room; after 15 min, the baseline variables were measured. During the experiment, they remained seated and rested for the first 120 min following each meal. The room temperature and humidity were maintained at 25 °C ± 2 °C and 50% ± 9%, respectively.

### 2.3. Measurements

#### 2.3.1. Physical Activity and Sleep/Wake Schedule

To evaluate spontaneous physical activity (steps), sleep/wake schedule (total sleep time, bedtime, and wake-up time), and pulse rate, we utilized the commercial sleep-tracking device Fitbit Inspire 2 (Fitbit Inc., San Francisco, CA, USA), which was worn on the participants’ left hand throughout the study period.

#### 2.3.2. Assessment of Blood Glucose, Plasma Insulin, and Plasma FFA Levels in the Capillary

We collected participants’ capillary blood samples at 8:00 (i.e., baseline), 9:00, 12:00 (immediately before lunch), 13:00, and 16:00 by pricking the index and middle fingers of their left hand. Capillary blood glucose concentrations were analyzed using GLUCOCARD PlusCare GT-1840 (Arkray, Kyoto, Japan). After collecting the blood samples into multiple 75 µL capillary tubes containing ethylenediaminetetraacetic acid as a blood anticoagulant, we separated the plasma (approximately 150 µL in total) by centrifugation at approximately 10,000–12,000× *g* for 5 min at room temperature and stored it at −80 °C until use. Capillary plasma insulin concentrations were measured using an enzyme immunoassay kit (Mercodia Insulin ELISA, Mercodia, Uppsala, Sweden). Next, FFA and triglyceride concentrations, as well as fasting total and high-density lipoprotein (HDL) cholesterol levels, from the capillary plasma samples were measured using the LabAssay kit (FUJIFILM Wako Shibayagi Co., Gunma, Japan).

#### 2.3.3. Continuous Glucose Monitoring (CGM)

To evaluate meal timing in free-living trials, we instructed all participants to wear a CGM system (FreeStyle Libre Pro; Abbott Laboratories, Chicago, IL, USA) to continuously track glucose levels throughout the trial. Once applied, the system can measure glucose levels at 15 min intervals for up to 14 days. The sensor was placed on the back of the upper arm 2 days before the start of the main protocol. Since this CGM system requires data to be read at least once every 8 h, otherwise earlier readings may be lost, participants were instructed to read their CGM data at five designated time points each day—immediately upon waking, before breakfast, before lunch, before dinner, and right before going to bed.

After the protocol was completed, the recorded data were subsequently downloaded from the CGM reader to a PC and analyzed using the FreeStyle LibreView software application (FreeStyle LibreLink). CGM readings were calibrated to capillary finger-prick blood glucose measurements obtained at 8:00 on days 1, 2, 5, 8, and 9, when fasting glucose levels are relatively stable. Specifically, early morning CGM values were adjusted to match the corresponding finger-prick measurements to correct for occasional discrepancies, such as instances in which the CGM recorded unexpectedly low glucose levels while the finger-prick measurements were within the normal range. We evaluated the peak value 2 h after each meal. Additionally, the incremental area under the curve (iAUC) for postprandial blood glucose levels, calculated from 30 min before each meal to 2 h after each meal, was determined using the trapezoidal rule. We used the average blood glucose concentration from midnight to 7:00 as an index of nocturnal (or sleep) blood glucose levels.

#### 2.3.4. Circulatory Measurements

At 7:45 on days 1, 2, 5, and 9, the heart rate and blood pressure were monitored using an electrocardiogram and an automatic sphygmomanometer (oscillometric method), respectively (DYNASCOPE DS-8100; Fukuda Denshi Co., Tokyo, Japan). Using a pulse-echo Doppler ultrasound (Aplio 300; Toshiba Medical Systems Co. Ltd., Tochigi, Japan), we measured beat-by-beat blood velocity from the right brachial artery to the distal third of the right inactive upper limb. This ultrasound device had a linear 11.0 MHz probe with an insonation angle below 60° to evaluate shear stress (the frictional force of blood flow to the wall of conduit arteries) associated with endothelium modulation. The sample volume (i.e., region of interest) was positioned at the center of the brachial artery and adjusted to cover the vessels’ full diameter. To calculate the second-by-second blood velocities of antegrade, retrograde, and mean, we sampled the Doppler signals for antegrade and retrograde flow and electrocardiography signals digitally at 20 kHz using an A/D converter (PowerLab 8/30, ADInstruments, Sydney, Australia) and analyzed them using the Doppler signal processing software (fast Fourier transfer analysis), following an established method used in our previous studies [22,25]. B-mode echo images of the right brachial artery were recorded simultaneously using a hard disk drive video recorder. In line with our previous study [22], the recorded B-mode images were converted into MPEG4 and captured at 30 Hz using a capture box (VirtualDubMod, 960 × 720-pixel resolution). The captured images were processed and analyzed using the Image-J software (Image-J Fiji). Initially, the region of interest was identified on the first frame of each participant. The luminance in the selected region of interest was output as 0 (black)–255 (white) values. In the B-mode echo images of the right brachial artery, the lumen and intima of the vessel were displayed in black and white, respectively. The vascular intima corresponded to a value with a rapid increase in luminance. To evaluate the blood vessel diameter, we first detected the intima of the anterior and posterior walls from the center of the region of interest, followed by the vessel diameter in the region of interest at 30 frames per second using an image processing algorithm based on the Visual Basic for Applications macro (Microsoft Excel 2019; Microsoft Press, Div. of Microsoft Corp., Redmond, WA, USA). To exclude the influence of arterial compliance in brachial artery diameter assessment, we used end-diastolic data from the continuous brachial artery diameter data over the cardiac cycle. The brachial artery diameter data were calculated as 1 s values by smoothing with 60 Hz as a bin and overlapping at 30 Hz. The image resolution was below 0.04 mm/pixel. According to a previous study [26], the shear rate (SR) was calculated as follows: SR = 4 × mean blood velocity/vessel diameter.

#### 2.3.5. VEF

VEF of the right brachial artery was evaluated by measuring FMD using ultrasound while participants were in the supine position on a bed at 7:45–7:55, immediately before fingertip blood collection. Given that body movement during blood collection reduces the FMD measurement accuracy, we avoided fingertip blood collection during FMD measurements. We evaluated the brachial artery diameter and mean blood velocity for 1 min at baseline, 5 min during cuff inflation in the right lower arm (i.e., distal to the epicondyles) (200 mmHg), and 3 min after deflation. To determine the postdeflation peak diameter, we used a Microsoft Excel Visual Basic for Applications macro. The percentage increase in the peak diameter from the mean baseline diameter over 1 min before cuff inflation indicated FMD. The SR area under the curve (SR_AUC_) was quantified as the area from deflation time to diameter peak time. We used the trapezoidal rule every 1 s to calculate the SR_AUC_. In accordance with a previously recommended method of FMD [3,4], FMD was divided by SR_AUC_ (FMD/SR_AUC_) to normalize FMD for eliciting a shear stimulus because FMD is affected by the postdeflation increase in shear stress. We also calculated the absolute changes in FMD and FMD/SR_AUC_ from the baseline values (ΔFMD and ΔFMD/SR_AUC,_ respectively) to determine the patterns of VEF change from baseline (Day 1).

### 2.4. Statistical Analysis

Data are presented as mean and standard deviation. The Shapiro–Wilk test for normality was first used for data validation. Step counts and sleep parameters were analyzed using one-way repeated-measures analysis of variance (ANOVA), and Tukey’s post hoc test was performed when a significant main effect was observed. The time and trial effects on body weight, BMI, HOMA-IR, blood pressure, heart rate, and diameter, SR, and FMD indices in brachial artery, capillary blood glucose, plasma insulin, and plasma FFA and triglycerides were tested using a two-way repeated analysis of variance (ANOVA). When a significant effect of interaction (i.e., trial × time) was detected, we conducted Dunnett’s post hoc test and Turkey’s postdoc test to determine the effects of time (change from Day 1) and trial, respectively. The relationships between blood data and FMD indices were evaluated using Pearson’s correlation coefficient. A two-tailed *p*-value ≤ 0.05 was considered statistically significant. All statistical data were analyzed using the SPSS PASW 18 statistical software (SPSS Inc., Chicago, IL, USA).

## 3. Results

### 3.1. Body Composition, Physical Activity, and Sleep

Body weight and BMI under fasting conditions before breakfast showed no significant differences among the three groups on days 1, 5, and 9 (*p* = 0.895 and 0.949, respectively) (Table 2). During the intervention from days 1 to 9, the average values of step counts, and sleep duration, wake-up time, and bedtime did not significantly differ between such groups (*p* = 0.200, 0.896, 0.937, and 0.809, respectively) (Table 3).

### 3.2. Blood Glucose and Plasma Insulin Levels in Capillaries

**Figure 2:**
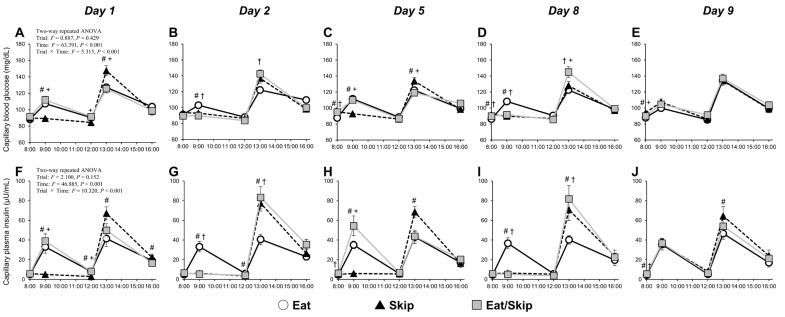
Time course of capillary blood glucose (**A**–**E**) and capillary plasma insulin (**F**–**J**) levels before and after breakfast consumption or skipping, followed by lunch on 5 experimental days (days 1, 2, 5, 8, and 9). White circles and solid lines, black triangles and dotted lines, and gray squares and solid lines represent the mean values for the Eat, Skip, and Eat/Skip trials, respectively. All data are presented as mean ± SD. ^#^ *p* < 0.05 Skip vs. Eat; ^†^ *p* < 0.05 Eat/Skip vs. Eat; ^+^ *p* < 0.05 Skip vs. Eat/Skip.

The two-way repeated measures ANOVA revealed a significant interaction effect (time × trial) on blood glucose and plasma insulin in capillaries (both: *p* < 0.001) (Figure 2). On days 1 and 5, at 8:00 (fasting), the Skip and Eat/Skip (eat day) trials had higher levels of capillary blood glucose than the Eat trial on Day 5 (Figure 2A,C). At 9:00 (30 min after breakfast), the levels were lower in the Skip trial than in the Eat and Eat/Skip (eat day) trials. At 13:00 (30 min after lunch), they were higher in the Skip trial than in the Eat and Eat/Skip (eat day) trials. On days 2 and 8, at 8:00 (fasting), the Skip and Eat/Skip trials (skip day) had higher levels of capillary blood glucose than the Eat trial on Day 8 (Figure 2B,D). At 9:00, the levels were lower in the Skip and Eat/Skip (skip day) trials than in the Eat trial. At 13:00, they were higher in the Eat/Skip (skip day) trial than in the Eat trial on Day 2 and in both the Eat and Skip trials on day 8. On Day 9, when all trials consumed breakfast at 8:00, the Skip trial had higher levels of capillary blood glucose before breakfast than the Eat and Eat/Skip trials (Figure 2E). Notably, we noted no significant differences between the trials after breakfast and lunch.

At 8:00 on days 1 and 5, the Eat/Skip (eat day) trial had higher levels of capillary plasma insulin on Day 5 than the Eat trial (Figure 2F,H). At 9:00 (30 min after breakfast), the levels were lower in the Skip trial than in the Eat trial and Eat/Skip (eat day) trials. Conversely, at 13:00 (30 min after lunch), they were higher in the Skip trial than in the Eat trial and tended to be higher than those in the Eat/Skip (eat day) trial (day 1: *p* = 0.056, Day 5: *p* = 0.002). At 8:00 on days 2 and 8, the capillary plasma insulin levels did not differ significantly between the three trials (Figure 2G,I). At 9:00, the levels were lower in the Skip and Eat/Skip (skip day) trials than in the Eat trial. At 13:00, they were higher in the Skip and Eat/Skip (skip day) trials than in the Eat trial. On Day 9, when all trials consumed breakfast at 8:00, the capillary plasma insulin levels before breakfast were significantly higher in the Skip and Eat/Skip trials than in the Eat trial (Figure 2J). Although no significant differences were observed among three trials after breakfast, the levels were significantly higher in the Skip trial than in the Eat trial after lunch.

### 3.3. CGM

The two-way repeated measures ANOVA revealed significant interactions for interstitial glucose dynamics, including peak glucose responses and the 2 h AUC after breakfast and lunch, as well as the mean glucose concentration during sleep (all *p* < 0.001) (Table 4). On days 1 and 5, the peak glucose levels after breakfast were lower in the Skip trial than in the Eat/Skip (eat day) trial and tended to be lower than in the Eat trial (Day 5: *p* = 0.079). On days 2 and 8, the levels were lower in both the Skip and Eat/Skip (skip day) trials than in the Eat trial. On Day 9, however, when all trials consumed breakfast at 8:00, they were significantly higher in the Skip trial than in the Eat and Eat/Skip trials. The iAUC8:30–10:30 after breakfast was lower in the Skip trial than in the other trials on days 1 and 5 and lower in both the Skip and Eat/Skip (skip day) trials than in the Eat trial on days 2 and 8. On Day 9, when all trials consumed breakfast at 8:00, no significant differences were observed between such trials. Regarding postlunch glycemic responses, the Skip trial showed the highest peak glucose levels on Day 1. A similar trend was observed on Day 5 (Eat: *p* = 0.086; Eat/Skip (eat day): *p* = 0.074). On days 2 and 8, the levels were significantly higher in the Eat/Skip (Skip day) trial than in the Eat trial, and on Day 8, they were also higher in the Eat/Skip trial than in the Skip trial. We observed no differences between the trials on Day 9.

The iAUC12:30–14:30 after lunch was higher in the Skip trial than in the other trials on days 1 and 5. The Eat/Skip (Skip day) trial exhibited significantly higher values than the Eat trial on days 2 and 8 and tended to be higher than the Skip trial on Day 8 (*p* = 0.089). On Day 9, when all trials consumed breakfast at 8:00, no significant differences were observed. Furthermore, the nocturnal glucose levels were significantly higher in the Skip trial than in the Eat trial on days 5, 8, and 9. The Eat/Skip trial on days 5 (eat day) and 8 (skip day) also showed higher levels than the Eat trial.

### 3.4. Plasma FFA and Triglyceride Levels in the Capillaries

**Figure 3:**
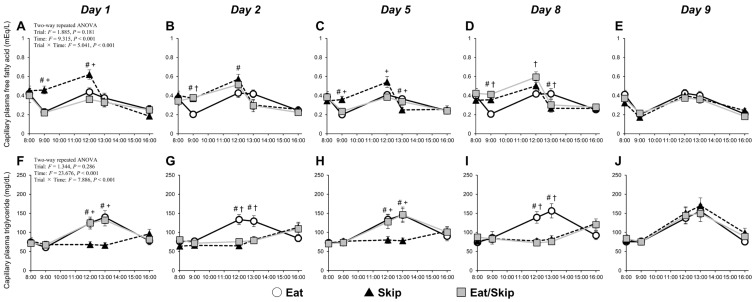
Time course of capillary plasma free fatty acid (**A**–**E**) and capillary plasma triglyceride (**F**–**J**) levels before and after breakfast consumption or skipping, followed by lunch on 5 experimental days (Day 1, 2, 5, 8, and 9). White circles and solid lines, black triangles and dotted lines, and gray squares and solid lines represent the mean values for the Eat, Skip, and Eat/Skip trials, respectively. All data are presented as mean ± SD. ^#^ *p* < 0.05 Skip vs. Eat; ^†^ *p* < 0.05 Eat/Skip vs. Eat; ^+^ *p* < 0.05 Skip vs. Eat/Skip.

The two-way repeated measures ANOVA revealed a significant interaction effect (time × trial) on capillary plasma FFA and triglycerides (both: *p* < 0.001). On days 1 and 5, at 9:00 (30 min after breakfast) and 12:00 (before lunch), the capillary plasma FFA levels in the Skip trial were higher than those in the Eat/Skip (eat day) trial; they were also higher or tended to be higher than those in the Eat trial (Day 1: *p* = 0.011, Day 5: *p* = 0.091) (Figure 3A,C). At 13:00 (30 min after lunch) on Day 5, the levels were lower in the Skip trial than in the Eat and Eat/Skip (eat Day) trials. At 9:00 on days 2 and 8, they were higher in the Skip and Eat/Skip (skip day) trials than in the Eat trial (Figure 3B,D). At 13:00 on Day 8, the levels were lower in the Skip and Eat/Skip (skip day) trials than in the Eat trial. On Day 9, breakfast was consumed at 8:00 in all trials. No significant differences in the capillary plasma FFA levels were observed among the three trials before and after breakfast or before and after lunch (Figure 3E).

At 12:00 (pre-lunch) and 13:00 (30 min postlunch) on days 1 and 5, the Skip trial had lower levels of capillary plasma triglycerides than the Eat and Eat/Skip (eat day) trials (Figure 3F,H). At 12:00 (pre-lunch) and 13:00 (30 min postlunch) on days 2 and 8, the levels were lower in the Skip and Eat/Skip (skip day) trials than in the Eat trial (Figure 3G,I). On Day 9, breakfast was consumed at 8:00 in all trials. The capillary plasma triglyceride levels did not significantly differ among the three trials before and after breakfast or before and after lunch (Figure 3J).

### 3.5. Fasting HOMA-IR, Total Cholesterol, and HDL Cholesterol

The two-way repeated measures ANOVA did not reveal a significant interaction effect (time × trial) on HOMA-IR (*p* = 0.358), ΔHOMA-IR (*p* = 0.358), total cholesterol (*p* = 0.481), and HDL cholesterol (*p* = 0.663) but showed a significant main effect of the trials on HOMA-IR (*p* = 0.003) (Table 5). On Day 1, HOMA-IR data did not significantly differ among the three trials (*p* > 0.05). The Skip trial showed higher HOMA-IR levels than the Eat trial on days 8 and 9 (*p* = 0.036 and *p* < 0.001, respectively). On Day 5, the Eat/Skip trial showed higher HOMA-IR levels (*p* = 0.004). These results seemed to reflect a slight (though not significant) decrease in HOMA-IR only in the Eat trial, with no significant changes in the Skip or Eat/Skip trials.

### 3.6. Heart Rate and Blood Pressure, Brachial Artery SR, Diameter, and VEF (FMD and FMD/SR_AUC_)

The two-way repeated measures ANOVA interaction (time and trial) revealed no significant effect on heart rate (*p* = 0.888), systolic blood pressure (*p* = 0.072) and diastolic blood pressure (*p* = 0.539). (Table 6).

**Figure 4:**
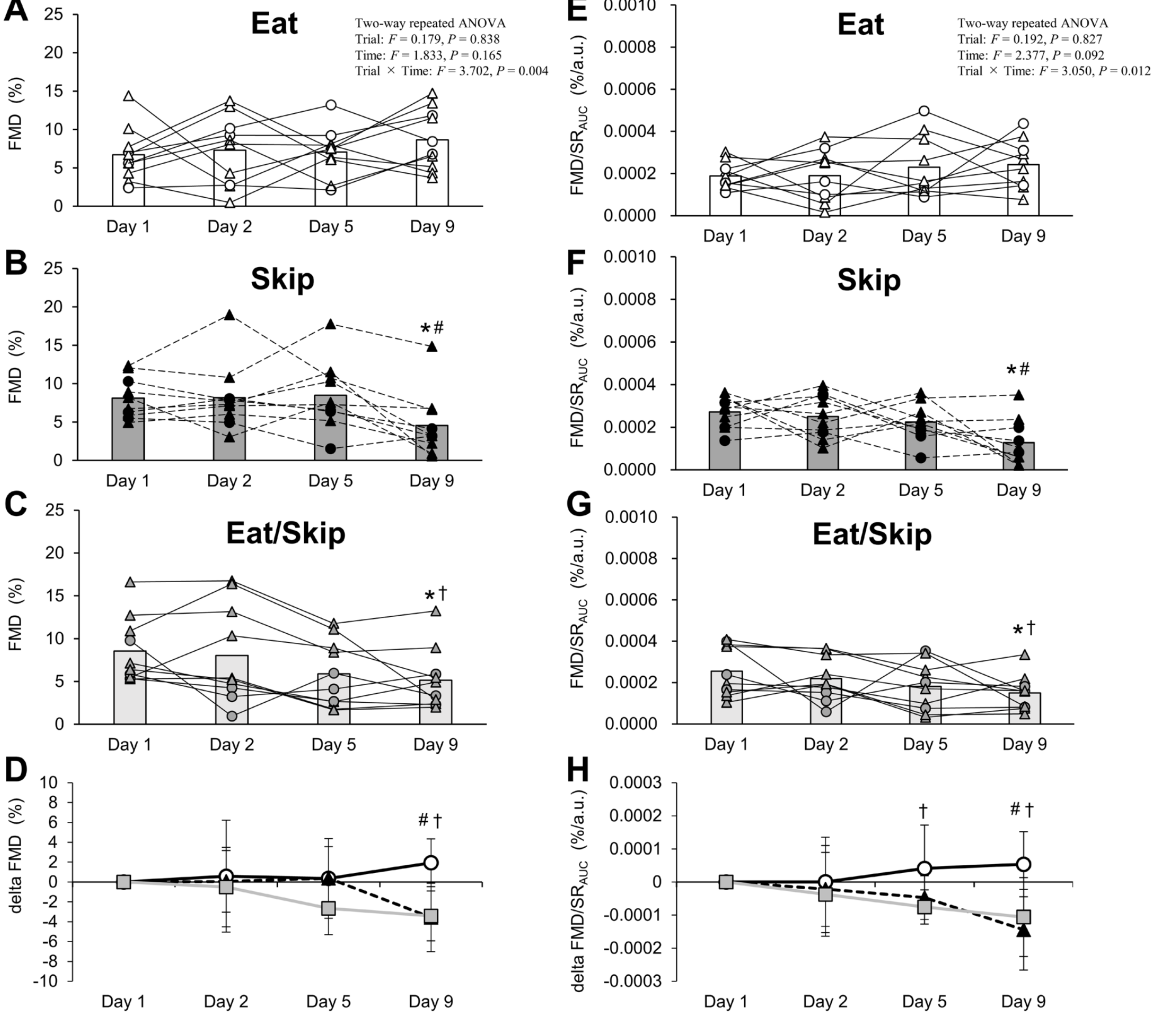
Time course of the relative change in brachial artery flow-mediated dilation (FMD) (**A**–**D**) and shear rate (SR)-corrected brachial artery FMD (**E**–**H**) before and after breakfast consumption or skipping, followed by lunch on 5 experimental days (days 1, 2, 5 and 9). Panels (**A**,**E**) (white shapes and solid lines), (**B**,**F**) (black shapes and dotted lines), and (**C**,**G**) (gray shapes and solid lines) show individual data points and mean values (bars) for the Eat, Skip, and Eat/Skip trials, respectively. The triangles and circles represent data from the female and male participants, respectively. Panels (**D**,**H**) show the time course of changes in FMD and FMD/SR_AUC_ from baseline (Day 1 at 8:00) as mean ± SD in all trials. * *p* < 0.05 vs. Day 1 (baseline) for Skip and Eat/Skip trials; ^#^ *p* < 0.05 Skip vs. Eat; ^†^ *p* < 0.05 Eat/Skip vs. Eat. SR_AUC_, shear rate area under the curve.

A two-way repeated measures ANOVA (factors: time and trial) revealed no significant interaction effects on the SR (*p* = 0.565), baseline diameter (*p* = 0.918), peak diameter (*p* = 0.143), time to peak diameter (*p* = 0.715), or the SR_AUC_ (*p* = 0.366) (Table 6). Conversely, the interaction effects for %FMD (*p* = 0.004), %FMD/SR_AUC_ (*p* = 0.012), change in %FMD (Δ%FMD; *p* = 0.004), and change in %FMD/SR_AUC_ (Δ%FMD/SR_AUC_) were significant (*p* = 0.012) (Table 6; Figure 4). At 8:00 on Day 1 (baseline), %FMD and %FMD/SR_AUC_ did not significantly differ among the three trials (*p* > 0.05; Figure 4A–C,E–G). However, both were significantly reduced in the Skip and Eat/Skip trials on Day 9 compared with those on Day 1 (*p* < 0.05; Figure 4B,C,F,G). Additionally, on Day 9, %FMD, Δ%FMD, %FMD/SR_AUC_, and Δ%FMD/SR_AUC_ were significantly lower in the Skip and Eat/Skip trials than in the Eat trial (*p* < 0.05; Figure 4A–H).

### 3.7. Relationship Between Changes in VEF and Fasting Blood Variable from Days 1 to 9

**Figure 5:**
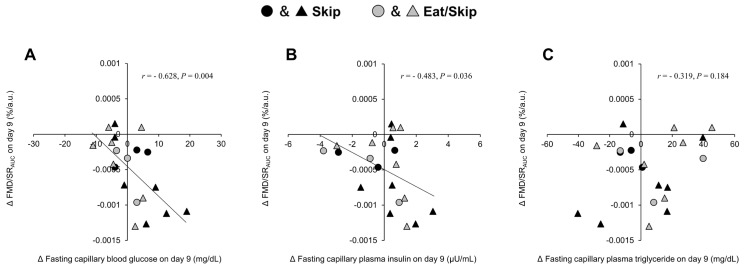
Correlations between changes in %FMD/SR_AUC_ and metabolic variables on Day 9: (**A**) Δfasting capillary blood glucose, (**B**) Δfasting plasma insulin, and (**C**) Δfasting plasma triglyceride. Analyses were performed using data from the Skip and Eat/Skip trials only, as no significant changes in FMD indices were observed in the Eat trial. Black, and gray symbols represent the Skip, and Eat/Skip trials, respectively. The triangles and circles within each trial represent data from female and male participants, respectively. FMD, flow mediated dilation; SR_AUC_, shear rate area under the curve.

Because FMD indices changed only during the Skip and Eat/Skip trials, correlation analyses were performed excluding the Eat trial data. This exclusion was made because no significant changes in FMD were observed in the Eat trial, and inclusion of this condition would likely attenuate the strength of associations between metabolic and vascular responses. Under these conditions, a significant correlation was observed between changes in fasting capillary blood glucose (*r* = −0.628, *p* = 0.004; *n* = 20) and fasting capillary plasma insulin (*r* = −0.483, *p* = 0.036; *n* = 20) with Δ%FMD/SR_AUC_ on Day 9 (Figure 5A,B), but not with Δ%FMD. Conversely, the change in fasting triglycerides on Day 9 did not significantly correlate with either Δ%FMD (*r* = 0.315, *p* = 0.188; *n* = 20) or Δ%FMD/SR_AUC_ (*r* = 0.319, *p* = 0.184; *n* = 20) (Figure 5C).

## 4. Discussion

This study is the first to examine the effects of different breakfast consumption patterns (daily consumption [Eat], daily skipping [Skip], and alternate-day skipping [Eat/Skip]) over an eight-day period on VEF in the brachial artery. The VEF was significantly reduced by repeated breakfast skipping for eight consecutive days compared with that by regular breakfast consumption. Notably, even with only four instances of breakfast skipping, the alternate-day pattern (Eat/Skip) also resulted in decreased VEF; hence, not only the frequency but also the irregularity of breakfast intake may negatively affect vascular health. Furthermore, in the Skip and Eat/Skip trials, fasting blood glucose and plasma insulin showed a moderate negative correlation with FMD/SR_AUC_, suggesting a potential link between metabolic status and vascular function under conditions of breakfast skipping. Additionally, on breakfast-skipping days under the Eat/Skip trial, postprandial hyperglycemia consistently occurred at lunch. However, by Day 8, in the Skip trial, the glycemic response was attenuated despite the same meal pattern, suggesting that the body had adapted metabolically to repeated breakfast skipping. These findings suggest that both the consistency and regularity of breakfast intake are essential in maintaining vascular endothelial health.

In the Eat/Skip trial, postprandial glycemic responses after lunch were consistently higher on breakfast-skipping days than on breakfast-consumed days (Eat trial), consistent with previous reports [13,14,15,22]. Conversely, postlunch glycemic responses clearly elevated on days 1 and 5 in the Skip trial compared with those in the Eat trial; however, by Day 8, despite a greater total energy intake in the Skip trial, the postlunch glucose elevation was nearly equivalent between the two trials. This observation supports the findings of Ogata et al. [23], who rigorously controlled meal quantity and timing. Interestingly, on Day 8, even under similar trials of breakfast skipping and high lunch volume, the glycemic response was lower in the Skip trial than in the Eat/Skip trial; thus, repeated exposure to skipping breakfast and/or excessive lunch intake may induce metabolic adaptations. Currently, the precise mechanisms underlying these adaptations remain unclear. Nonetheless, given the absence of a significant difference between the Skip and Eat/Skip trials in insulin levels at 30 min postlunch on Day 8, repeated breakfast skipping may improve insulin sensitivity in peripheral tissues. Supporting this notion, a cross-sectional study by Thomas et al. [27] reported that postlunch following breakfast skipping glycemic responses were lower in individuals who habitually skip breakfast than in habitual breakfast eaters. Therefore, insulin sensitivity in peripheral tissues can potentially be remodeled according to habitual meal-skipping patterns and habitual meal size. Taken together, following a consistent pattern of daily breakfast skipping combined with a large lunch intake may initially impair glycemic control but could promote metabolic adaptation over time. Conversely, alternating between breakfast consumption and skipping (Eat/Skip) appears less likely to trigger such adaptive changes. Furthermore, the average nocturnal glucose levels (00:00–07:00) were consistently higher in the Skip trial than in the Eat trial from Day 3 onward, possibly reflecting increased macronutrient intake at dinner under the Skip trial and/or a reduction in nocturnal insulin secretion or sensitivity [28,29].

Breakfast consumption promotes insulin secretion, consequently reducing plasma FFA concentrations. When breakfast is skipped, the FFA levels tend to increase slightly before lunch and then rapidly decrease after lunch. Such postprandial FFA dynamics, which were observed in the present study, are generally consistent across studies [13,14,22]. Notably, pre-lunch FFA elevation following breakfast skipping is reportedly one of the contributors to postprandial hyperglycemia via glucose uptake inhibition in peripheral tissues [13]. This study revealed significantly higher postprandial glycemic and insulin responses after lunch in the Skip trial than in the Eat trial; hence, skipping breakfast may inhibit glucose uptake associated with reduced insulin sensitivity at lunch, consistent with previous study results [14,22]. Regarding glucose uptake inhibition in peripheral tissues, residual FFA levels after skipping breakfast are an important factor in inducing postprandial hyperglycemia. According to the study of Kim et al. [20,21], FFAs promote serine phosphorylation of the insulin receptor substrate (IRS) and downregulate insulin signaling related to tyrosine phosphorylation of the IRS, phosphatidylinositol 3-kinase (PI3-kinase), phosphoinositide-dependent kinase, and Akt, consequently reducing glucose transport into cells.

Interestingly, on Day 8 of the intervention, despite that pre-lunch FFA concentrations did not significantly differ between the Skip and Eat/Skip trials, only the Eat/Skip trial exhibited postprandial hyperglycemia. Therefore, repeated breakfast skipping may lead to a metabolic rhythm adaptation, thereby enhancing metabolic flexibility. Metabolic flexibility is the body’s ability to efficiently switch between lipid and carbohydrate metabolism in response to nutrient availability—a hallmark of metabolic health [30]. In the Skip trial, the metabolic environment might have been reorganized temporarily following continuous breakfast skipping, potentially enhancing insulin sensitivity during the day’s first meal.

On Day 9, all participants from all trials received the same breakfast and lunch. While postprandial FFA or triglyceride concentrations showed no significant difference between trials, the peak interstitial glucose concentration after breakfast was significantly higher in the Skip trial than in the Eat and Eat/Skip trials. This result aligns with a previous finding [27], which reported greater glycemic responses among breakfast skippers when reintroduced to breakfast. Although postlunch glucose responses did not differ significantly among trials, the Skip trial had significantly higher insulin secretion than the Eat trial, suggesting postprandial insulin resistance following breakfast skipping. This finding partially corresponds to those of Farshchi et al. [31], who observed greater insulin responses in participants who resumed breakfast after 14 days of skipping. Thus, in the Skip trial, the metabolic adaptations established over 8 consecutive days of breakfast skipping may have been disrupted upon reintroducing breakfast on Day 9, leading to impaired glucose regulation. In the present study, skipped breakfast was reintroduced on Day 9 to standardize energy intake across trials. However, for weight-loss interventions aiming to maintain a hypocaloric diet, reintroduction of skipped meals should be avoided. Under such conditions, postprandial metabolic and vascular responses may differ from those observed in the current study.

In this study, both %FMD and %FMD/SR_AUC_ were significantly lower in the Skip trial than in the Eat trial and further decreased on Day 9 relative to baseline. Therefore, repeated postprandial hyperglycemia at lunch following breakfast skipping may contribute to sustained VEF suppression. Postprandial hyperglycemia has been reported to elevate reactive oxygen species production, acutely impairing the VEF of brachial artery [16,17,18]. Moreover, FMD further decreases in cases wherein larger postlunch glucose responses occurred after breakfast skipping [16]. Noteworthily, on Day 8 of the intervention, postprandial glucose responses in the Skip trial were comparable to those in the Eat trial, with no hyperglycemia observed, indicating metabolic adaptation resulting from repeated breakfast skipping. Therefore, further extending the intervention period could potentially lead to different effects on VEF. The higher fasting capillary blood glucose and plasma insulin in both breakfast skipping pattern trials during intervention may be partly associated with reductions in %FMD/SR_AUC_ observed in the Skip and Eat/Skip trials. These correlations do not include data from the Eat trial, as no significant FMD changes were observed there. Additionally, participants in the Skip and Eat/Skip (skip day) trials consumed consistently large evening meals despite timing of lower physical activity levels, possibly maintaining elevated glucose and insulin levels overnight; this effect may have contributed to impaired VEF.

Notably, FMD significantly decreased in the Eat/Skip trial. Although the number of skipped breakfasts was relatively low (4 days), the magnitude of FMD reduction was comparable to that in the continuous skipping trial; thus, irregular meal patterns, rather than the total frequency of breakfast skipping, may exert adverse effects on VEF. Furthermore, postlunch glucose levels on the skipping days (days 2 and 8) were consistently higher in the Eat/Skip trial than in the Eat trial, with no indication of metabolic adaptation seen in the continuous skipping group. Fasting blood glucose and plasma insulin also significantly elevated on certain days and tended to increase. Therefore, even with identical energy intake across trials, fluctuations in meal timing and quantity may exacerbate glucose metabolism. Indeed, epidemiological evidence indicates that insulin resistance indices are significantly higher in individuals with irregular eating patterns than in those who maintain regular meal schedules [32].

Triglyceride levels measured between 12:00 and 13:00 were consistently higher in the Eat trial than in both the Skip trial and the skipping days of the Eat/Skip trial; hence, breakfast consumption may influence postprandial triglyceride responses. While triglyceride levels declined after 13:00 in the Eat trial, they continued to rise in the two other trials; this effect is expected, given the higher fat intake at lunch in the two latter conditions. Although postprandial triglyceride levels were not measured after 16:00, postdinner triglyceride levels might be also elevated on the Skip trial and the skipping days of the Eat/Skip trial compared with those in the Eat trial, likely attributable to the additional consumption of granola containing 13.1 g of fat at dinner in the Skip and Eat/Skip trials. If postprandial triglyceride levels increase, fatty acids are oxidized within the endothelium, inducing local oxidative stress and subsequently reducing nitric oxide bioavailability, thereby contributing to endothelial dysfunction [33]. Additionally, postprandial lipid metabolism exhibits time-of-day dependency; when meals are consumed at night, triglyceride responses are heightened [34]. However, triglyceride levels after dinner were not assessed in this study, thereby warranting further investigation.

Moreover, fasting triglycerides, total cholesterol, and HDL cholesterol showed no significant differences between the trials. Conversely, Farshchi et al. [31] reported increases in total and LDL cholesterol after 14 days of breakfast omission, but only in the skipping condition. This discrepancy may be attributable to food intake differences during the intervention. In their study, food intake was greater in the skipping trial, whereas in our study, all meals were standardized in both quantity and composition across trials. These methodological differences may account for the divergent outcomes in fasting lipid levels.

This study has several limitations. First, postprandial blood samples were collected at only two time points (30 and 180 min after meals) based on our previous findings showing that most participants reached peak blood glucose concentrations at 30 min after a meal [22]. However, blood sampling was impossible after 16:00. Consequently, we could not assess lipid profile changes after lunch and dinner, thereby limiting our understanding of postprandial lipid metabolism throughout the day. Second, the dietary fiber content of the test meals was not precisely quantified. Because the meals were composed of commercially available prepackaged products, and dietary fiber information was not provided for several items, potential effects of dietary fiber on postprandial metabolism and vascular responses could not be fully evaluated. However, as the same meals were used across all experimental conditions, daily dietary fiber intake was identical between conditions, and therefore unlikely to have affected the observed differences in vascular responses. Another limitation is that the menstrual cycle phases of the female participants were not standardized. VEF is influenced by hormonal fluctuations throughout the menstrual cycle [35], and differences in cycle timing may have influenced the results. Our 10-day intervention period, including the day of dietary adjustment, made all tests within the early follicular phase (up to Day 8; when female hormone levels are relatively stable) difficult to complete, assuming that Day 0 corresponds to the menstruation onset. In contrast, the VEF in all three male participants decreased on Day 9 compared with that on Day 1 in both the Skip and Eat/Skip trials. Further investigation is needed to determine whether this trend is independent of sex differences. Moreover, our study has a small sample size; thus, we could not fully examine the sex-specific effects, which may be important in studies of vascular and metabolic function. All participants were also young and healthy, limiting the generalizability of these findings to older adults or those with metabolic or cardiovascular disease. Older adults or post-menopausal women often exhibit reduced metabolic adaptability, and breakfast skipping in these populations could potentially induce more pronounced metabolic and vascular impairments. Future studies are warranted to investigate age- and sex-specific responses to meal timing interventions. Furthermore, this study did not examine potential nocturnal effects or chronic adaptations, both of which may be important for understanding the long-term effects of meal timing and composition on vascular health. Finally, we did not assess endothelium-independent vasodilation; hence, we could not determine whether the observed changes in vascular function were exclusively endothelium-dependent. Therefore, further studies are warranted to address these limitations and elucidate the underlying mechanisms.

## 5. Conclusions

This study investigated the effects of either regular or irregular breakfast skipping on brachial artery VEF before and after an eight-day intervention. In both regular and irregular breakfast-skipping conditions, early morning fasting VEF was suppressed. Therefore, regular breakfast consumption is essential to preserve VEF and support cardiovascular health.

## Figures and Tables

**Table 1:** Five types of meals and their dietary compositions for females during the intervention.

Meal Type	Diet	Menu	Energy (kcal)	Protein (g)	Fat (g)	Carbohydrates (g)	Salt (g)
Breakfast	A	Cereal, milk, steamed chicken, and pumpkin soup	506	29.2	22.7	50.8	1.81
	B	Cereal, milk, steamed chicken, and tomato soup	492	29.1	22.6	47.3	1.81
	C	Cereal, milk, steamed chicken, and minestrone	464	27.8	21.9	42.1	2.01
	D	Cereal, milk, steamed chicken, and tomato soup	492	29.1	22.6	47.3	1.81
	E	Cereal, milk, steamed chicken, and mushroom soup and milk	475	28.6	22.2	44.5	1.71
Lunch	A	Spaghetti, pesto sauce, vegetable juice, and brown rice bran	729	19.9	22.5	114.3	5.37
	B	White rice, buttered chicken curry, vegetable juice, and brown rice bran	840	16.4	31.6	124.4	3.54
	C	Spaghetti, bolognese meat sauce, vegetable juice, and brown rice bran	843	31.1	27.3	120.7	2.94
	D	White rice, hashed beef, vegetable juice, and brown rice bran	719	17.4	20.4	118.3	2.74
	E	Spaghetti, carbonara sauce, vegetable juice, and brown rice bran	775	24.6	23.6	118.6	5.62
Dinner	A	Beef stew with red wine, white rice, and miso soup with vegetables	598	25.4	20.0	78.3	3.00
	B	Simmered yellowtail with white radish, white rice, and miso soup with vegetables	436	21.3	2.6	81.2	3.60
	C	Mapo tofu, white rice, and miso soup with vegetables	500	15.0	13.1	80.0	4.30
	D	Mackerel stew in miso, white rice, pumpkin soup, and miso soup with vegetables	624	25.0	15.3	97.2	3.80
	E	Hamburg steak, white rice, and miso soup with vegetables	589	20.1	18.8	84.1	3.00
Total	A	N/A	1833	74.5	65.2	243.4	10.18
	B	N/A	1768	66.8	56.8	252.9	8.95
	C	N/A	1807	73.9	62.3	242.8	9.25
	D	N/A	1835	71.5	58.3	262.8	8.35
	E	N/A	1839	73.3	64.6	247.2	10.33

Meals A, B, C, D, and E were served on the following days: A on the day before the experiment (preday) and on day 5; B on days 3 and 9; C on days 3 and 6; D on days 1 and 9; and E on days 4 and 7; N/A, not applicable.

**Table 2:** Body weight and body mass index before, during, and after the intervention. Data are presented as mean ± SD.

	Day 1	Day 5	Day 9	Two-Way Repeated Measures ANOVA
	(baseline)			
Height, cm				
Eat	160 ± 4	N/A	N/A	N/A
Skip	160 ± 4	N/A	N/A	N/A
Eat/Skip	160 ± 4	N/A	N/A	N/A
Body weight, kg				
Eat	54.1 ± 5.2	54.1 ± 5.5	54.1 ± 5.4	Trial: *F* = 1.316, *p* = 0.293
Skip	54.0 ± 5.1	53.9 ± 5.3	53.9 ± 5.1	Time: *F* = 0.003, *p* = 0.997
Eat/Skip	54.5 ± 5.3	54.5 ± 5.2	54.6 ± 5.4	Trial × Time: *F* = 0.271, *p* = 0.895
Body mass index, kg/m^2^				
Eat	21.1 ± 2.1	21.1 ± 2.1	21.1 ± 2.2	Trial: *F* = 1.220, *p* = 0.319
Skip	21.1 ± 2.1	21.1 ± 2.2	21.1 ± 2.1	Time: *F* = 0.006, *p* = 0.994
Eat/Skip	21.3 ± 2.2	21.3 ± 2.1	21.3 ± 2.2	Trial × Time: *F* = 0.177, *p* = 0.949

N/A, not applicable.

**Table 3:** Mean daily step count, sleep duration, wake up time, and bedtime during the 9-day intervention period. Data are presented as mean ± SD.

Mean Value from Days 1 to 9	Eat	Skip	Eat/Skip	One-Way ANOVA
Step (steps)	6565 ± 1336	5610 ± 2174	6501 ± 1640	*F* = 1.761, *p* = 0.200
Sleep duration (min)	377 ± 33	373 ± 33	378 ± 23	*F* = 0.111, *p* = 0.896
Wake up time (hh:mm)	6:31 ± 0:22	6:30 ± 0:23	6:32 ± 0:26	*F* = 0.066, *p* = 0.937
Bedtime (hh:mm)	23:18 ± 0:45	23:18 ± 0:42	23:24 ± 0:38	*F* = 0.214, *p* = 0.809

**Table 4:** Peak glucose levels and glucose AUC during waking hours and mean glucose levels during sleep, measured by continuous glucose monitoring for five experimental days. Data are presented as mean ± SD. ^#^ *p* < 0.05, Skip vs. Eat; ^†^ *p* < 0.05, Eat/Skip vs. Eat; ^+^ *p* < 0.05, Skip vs. Eat/Skip.

		Day 1	Day 2	Day 5	Day 8	Day 9	Two-Way Repeated ANOVA
		(baseline)					
Breakfast	Peak glucose response, mg/dL						
	Eat	107.1 ± 7.6	110.7 ± 11.7	115.5 ± 19.8	103.6 ± 5.7	108.8 ± 6.3	Trial: *F* = 12.518, *p* < 0.001
	Skip	94.1 ± 4.4 ^#,+^	97.9 ± 8.1 ^#^	102.4 ± 9.8 ^+^	94.3 ± 6.1 ^#^	117.9 ± 6.9 ^#,+^	Time: *F* = 9.416, *p* < 0.001
	Eat/Skip	116.2 ± 13.1	97.4 ± 10.6 ^†^	118.1 ± 9.8	96.3 ± 4.4 ^†^	109.7 ± 10.2	Trial × Time: *F* = 6.599, *p* < 0.001
	Glucose AUC _8:30–10:30_, mg/dL × min						
	Eat	977 ± 895	1149 ± 650	1412 ± 981	855 ± 480	1160 ± 782	Trial: *F* = 41.638, *p* < 0.001
	Skip	−466 ± 396 ^#,+^	−365 ± 343 ^#^	−292 ± 485 ^#,+^	−606 ± 719 ^#^	877 ± 481	Time: *F* = 10.962, *p* < 0.001
	Eat/Skip	1279 ± 7.8	7 ± 666 ^†^	1474 ± 663	−1 ± 621 ^†^	1115 ± 603	Trial × Time: *F* = 8.814, *p* < 0.001
Lunch	Peak glucose response, mg/dL						
	Eat	137.3 ± 17.3	130.5 ± 13.4	129.4 ± 19.3	124.9 ± 13.3	131.5 ± 18.3	Trial: *F* = 12.404, *p* < 0.001
	Skip	156.3 ± 16.1 ^#,+^	140.4 ± 11.4	145.8 ± 21.3	136.2 ± 13.1 ^+^	142.3 ± 14.3	Time: *F* = 1.376, *p* = 0.262
	Eat/Skip	138.0 ± 13.5	152.8 ± 21.6 ^†^	128.7 ± 16.4	154.5 ± 20.1 ^†^	142.8 ± 13.7	Trial × Time: *F* = 4.331, *p* < 0.001
	Glucose AUC _12:30–14:30_, mg/dL × min						
	Eat	3358 ± 1504	3812 ± 1447	3500 ± 1305	3804 ± 1330	4331 ± 1336	Trial: *F* = 8.488, *p* = 0.003
	Skip	6091 ± 1737 ^#,+^	4821 ± 1154	4946 ± 1875 ^#,+^	4357 ± 1490	3740 ± 1635	Time: *F* = 2.792, *p* = 0.041
	Eat/Skip	3544 ± 1860	5597 ± 2027 ^†^	3057 ± 1075	5506 ± 1787 ^†^	3890 ± 1617	Trial × Time: *F* = 6.046, *p* < 0.001
Dinner	Peak glucose response, (mg/dL)						
	Eat	155.0 ± 22.3	164.6 ± 28.3	149.6 ± 15.5	154.6 ± 28.5	165.7 ± 20.8	Trial: *F* = 1.175, *p* = 0.334
	Skip	149.4 ± 22.6	151.1 ± 19.1	154.3 ± 37.7	145.5 ± 26.9	174.9 ± 23.0	Time: *F* = 0.478, *p* = 0.752
	Eat/Skip	159.1 ± 27.0	156.1 ± 29.4	147.4 ± 23.7	155.5 ± 27.4	160.6 ± 18.9	Trial × Time: *F* = 1.088, *p* = 0.38
	Glucose AUC _20:00–22:00_, mg/dL × min						
	Eat	6267 ± 2846	6093 ± 2433	5326 ± 1826	5780 ± 2104	7022 ± 1954	Trial: *F* = 4.437, *p* = 0.029
	Skip	4488 ± 2002	4499 ± 2022	4721 ± 2085	4050 ± 2270	6411 ± 1958	Time: *F* = 4.277, *p* = 0.007
	Eat/Skip	4662 ± 1580	5202 ± 2204	4592 ± 1929	5123 ± 2267	5777 ± 1942	Trial × Time: *F* = 2.026, *p* = 0.057
Sleep	Mean glucose levels during sleep, mg/dL						
	Eat	N/A	90.6 ± 10.7	87.8 ± 7.1	82.2 ± 4.1	87.0 ± 5.8	Trial: *F* = 12.479, *p* < 0.001
	Skip	N/A	94.5 ± 8.7	95.8 ± 6.9 ^#^	92.6 ± 7.7 ^#^	95.4 ± 6.3 ^#^	Time: *F* = 4.330, *p* = 0.013
	Eat/Skip	N/A	90.9 ± 8.3	99.3 ± 5.5 ^#^	89.4 ± 6.4 ^†^	90.8 ± 4.1	Trial × Time: *F* = 3.313, *p* = 0.008

AUC, area under the curve. N/A, not applicable.

**Table 5:** HOMA-IR and fasting levels of total cholesterol and HDL-cholesterol during the five experimental days. Data are presented as mean ± SD. ^#^ *p* < 0.05, Skip vs. Eat; ^†^ *p* < 0.05, Eat/Skip vs. Eat.

	Day 1	Day 2	Day 5	Day 8	Day 9	Two-Way Repeated ANOVA
	(baseline)					
HOMA-IR						
Eat	1.27 ± 0.68	1.16 ± 0.60	1.09 ± 0.35	1.00 ± 0.35	0.97 ± 0.35	Trial: *F* = 7.985, *p* = 0.003
Skip	1.37 ± 0.36	1.43 ± 0.59	1.27 ± 0.37	1.33 ± 0.38 ^#^	1.45 ± 0.56 ^#^	Time: *F* = 0.487, *p* = 0.745
Eat/Skip	1.40 ± 0.44	1.51 ± 0.46	1.54 ± 0.51 ^†^	1.30 ± 0.43	1.24 ± 0.35	Trial × Time: *F* = 0.380, *p* = 0.358
Total-cholesterol, mg/dL						
Eat	171.8 ± 9.1	158.6 ± 11.1	165.9 ± 10.1	175.7 ± 10.9	173.8 ± 11.0	Trial: *F* = 2.405, *p* = 0.119
Skip	166.0 ± 8.8	181.0 ± 11.7	183.8 ± 13.9	184.2 ± 11.9	184.0 ± 11.6	Time: *F* = 1.231, *p* = 0.315
Eat/Skip	171.4 ± 8.6	159.2 ± 9.5	172.6 ± 12.3	183.0 ± 13.5	183.0 ± 14.4	Trial × Time: *F* = 0.951, *p* = 0.481
HDL-cholesterol, mg/dL						
Eat	55.3 ± 9.7	52.2 ± 9.9	52.8 ± 13.3	53.9 ± 9.3	52.4 ± 8.8	Trial: *F* = 0.824, *p* = 0.454
Skip	56.8 ± 14.0	58.8 ± 12.9	55.0 ± 15.3	55.9 ± 9.2	55.6 ± 13.7	Time: *F* = 0.238, *p* = 0.915
Eat/Skip	50.5 ± 9.4	53.8 ± 7.0	54.8 ± 12.0	57.4 ± 15.1	57.9 ± 11.6	Trial × Time: *F* = 0.731, *p* = 0.663

HDL, high-density lipoprotein; HOMA-IR, homeostasis model assessment of insulin resistance.

**Table 6:** Heart rate, blood pressure, brachial artery diameter, shear rate, and flow-mediated dilation indices measured in the early morning of four experimental days. Data are presented as mean ± SD. * p < 0.05 vs. Day 1 (baseline); ^#^ *p* < 0.05, Skip vs. Eat; ^†^ *p* < 0.05, Eat/Skip vs. Eat.

	**Day 1**	**Day 2**	**Day 5**	**Day 9**	**Two-Way Repeated ANOVA**
	(baseline)				
Heat rate, beats/min					
Eat	66.3 ± 12.2	66.7 ± 8.3	67.9 ± 7.4	66.0 ± 5.9	Trial: *F* = 1.741, *p* = 0.204
Skip	65.8 ± 9.7	67.2 ± 6.5	66.1 ± 6.3	66.5 ± 8.1	Time: *F* = 0.142, *p* = 0.934
Eat/Skip	66.1 ± 10.0	65.5 ± 9.9	63.1 ± 6.2	64.8 ± 10.3	Trial × Time: *F* = 0.380, *p* = 0.888
Systolic blood pressure, mmHg					
Eat	110.9 ± 6.8	112.6 ± 5.7	111.4 ± 7.2	110.0 ± 8.6	Trial: *F* = 0.304, *p* = 0.741
Skip	108.1 ± 8.5	111.7 ± 8.4	109.7 ± 9.1	110.8 ± 10.2	Time: *F* = 0.794, *p* = 0.508
Eat/Skip	108.9 ± 8.6	108.8 ± 7.9	108.8 ± 9.9	110.8 ± 10.3	Trial × Time: *F* = 2.071, *p* = 0.072
Diastolic blood pressure, mmHg					
Eat	59.3 ± 5.5	60.6 ± 5.8	59.9 ± 5.9	59.1 ± 5.7	Trial: *F* = 0.240, *p* = 0.789
Skip	58.8 ± 6.6	60.5 ± 6.1	57.6 ± 6.6	58.7 ± 7.5	Time: *F* = 1.291, *p* = 0.297
Eat/Skip	58.2 ± 6.4	58.8 ± 6.0	57.4 ± 7.2	59.2 ± 6.7	Trial × Time: *F* = 0.848, *p* = 0.539
Shear rate, s^−1^					
Eat	255.6 ± 125.7	295.6 ± 110.3	252.7 ± 116.4	243.8 ± 131.8	Trial: *F* = 0.383, *p* = 0.687
Skip	210.5 ± 80.2	247.4 ± 102.5	220.4 ± 95.1	245.2 ± 117.0	Time: *F* = 0.954, *p* = 0.428
Eat/Skip	233.4 ± 98.9	229.7 ± 84.6	240.0 ± 133.8	243.3 ± 105.1	Trial × Time: *F* = 0.813, *p* = 0.565
Baseline diameter, cm					
Eat	0.297 ± 0.029	0.297 ± 0.032	0.296 ± 0.026	0.302 ± 0.026	Trial: *F* = 0.049, *p* = 0.952
Skip	0.298 ± 0.032	0.295 ± 0.026	0.301 ± 0.027	0.306 ± 0.038	Time: *F* = 3.549, *p* = 0.028
Eat/Skip	0.301 ± 0.026	0.294 ± 0.035	0.302 ± 0.031	0.305 ± 0.035	Trial × Time: *F* = 0.331, *p* = 0.918
Peak diameter, cm					
Eat	0.317 ± 0.031	0.319 ± 0.038	0.318 ± 0.034	0.328 ± 0.031	Trial: *F* = 0.024, *p* = 0.976
Skip	0.322 ± 0.036	0.320 ± 0.033	0.326 ± 0.027	0.320 ± 0.037	Time: *F* = 0.628, *p* = 0.603
Eat/Skip	0.326 ± 0.025	0.316 ± 0.027	0.319 ± 0.025	0.320 ± 0.033	Trial × Time: *F* = 1.685, *p* = 0.143
Time to peak diameter, s					
Eat	66.6 ± 26.2	71.3 ± 21.7	61.3 ± 23.6	70.1 ± 18.4	Trial: *F* = 1.776, *p* = 0.198
Skip	58.0 ± 13.8	67.1 ± 24.6	65.1 ± 17.1	62.5 ± 14.8	Time: *F* = 0.470, *p* = 0.706
Eat/Skip	59.8 ± 15.1	56.6 ± 15.6	57.4 ± 14.3	60.7 ± 16.0	Trial × Time: *F* = 0.618, *p* = 0.715
SR_AUC_, a. u.					
Eat	36,007 ± 14,563	39,789 ± 11,913	34,731 ± 13,031	38,396 ± 12,452	Trial: *F* = 0.596, *p* = 0.561
Skip	30,426 ± 7493	34,016 ± 12,641	36,697 ± 9259	37,752 ± 13,856	Time: *F* = 0.669, *p* = 0.579
Eat/Skip	35,461 ± 8885	32,946 ± 10,288	35,836 ± 12,558	34,198 ± 10,206	Trial × Time: *F* = 1.114, *p* = 0.366
%FMD, %					
Eat	6.71 ± 3.51	7.30 ± 4.54	7.08 ± 3.15	8.65 ± 3.96	Trial: *F* = 0.179, *p* = 0.838
Skip	8.12 ± 2.71	8.19 ± 4.31	8.49 ± 4.40	4.57 ± 4.16 *^,#^	Time: *F* = 1.833, *p* = 0.165
Eat/Skip	8.56 ± 3.86	8.04 ± 5.69	5.90 ± 3.89	5.14 ± 3.57 *^,†^	Trial × Time: *F* = 3.702, *p* = 0.004
%FMD/SR_AUC_, %/a.u.					
Eat	0.00019 ± 0.00006	0.00019 ± 0.00012	0.00023 ± 0.00014	0.00024 ± 0.00011	Trial: *F* = 0.192, *p* = 0.827
Skip	0.00027 ± 0.00007	0.00025 ± 0.00010	0.00022 ± 0.00009	0.00013 ± 0.00011 *^,#^	Time: *F* = 2.377, *p* = 0.092
Eat/Skip	0.00026 ± 0.00012	0.00022 ± 0.00011	0.00018 ± 0.00012	0.00015 ± 0.00009 *^,†^	Trial × Time: *F* = 3.050, *p* = 0.012
Δ%FMD, %					
Eat	N/A	0.59 ± 5.64	0.36 ± 4.02	1.93 ± 2.41	Trial: *F* = 3.755, *p* = 0.043
Skip	N/A	0.06 ± 3.45	0.37 ± 3.19	−3.56 ± 3.45 *^,#^	Time: *F* = 1.833, *p* = 0.165
Eat/Skip	N/A	−0.51 ± 4.00	−2.7 ± 2.65	−3.42 ± 2.51 *^,†^	Trial × Time: *F* = 3.702, *p* = 0.004
Δ%FMD/SR_AUC_, %/a.u.					
Eat	N/A	0.00000 ± 0.00014	0.00004 ± 0.00013	0.00005 ± 0.00010	Trial: *F* = 5.932, *p* = 0.010
Skip	N/A	−0.00002 ± 0.00013	−0.00005 ± 0.00007	−0.00014 ± 0.00012 *^,#^	Time: *F* = 2.377, *p* < 0.092
Eat/Skip	N/A	−0.00003 ± 0.00012	−0.00008 ± 0.00005 ^†^	−0.00011 ± 0.00012 *^,†^	Trial × Time: *F* = 3.050, *p* = 0.012

a.u., arbitrary units; FMD, flow mediated dilation; N/A, not applicable; SR_AUC_, shear rate area under the curve.

## Data Availability

Data available on request due to restrictions, e.g., privacy or ethical, from the corresponding author.

## Abbreviations

The following abbreviations are used in this manuscript:

ANOVA	Analysis of Variance
BMI	Body Mass Index
CGM	Continuous Glucose Monitoring
FFA	Free Fatty Acid
FMD	Flow-Mediated Dilation
HDL	High-Density Lipoprotein
HOMA-IR	Homeostasis Model Assessment of Insulin Resistance
iAUC	Incremental Area Under the Curve
SR	Shear Rate
SR_AUC_	Shear Rate Area Under the Curve
VEF	Vascular Endothelial Function
